# *In vitro* examination of starch digestibility of Saba banana [*Musa ‘saba’*(*Musa acuminata* × *Musa balbisiana*)]: impact of maturity and physical properties of digesta

**DOI:** 10.1038/s41598-020-58611-5

**Published:** 2020-02-04

**Authors:** Florencio Collado Reginio, Sunantha Ketnawa, Yukiharu Ogawa

**Affiliations:** 1 Graduate School of Horticulture, Chiba University, 648 Matsudo, Matsudo City, Chiba 271-8510 Japan; 2 Institute of Food Science and Technology, College of Agriculture and Food Science, University of the Philippines Los Baños, College, Laguna 4031 Philippines

**Keywords:** Plant sciences, Nutrition

## Abstract

The digestibility of starch in Saba banana as affected by maturity and physical properties of digesta was investigated. Five maturity stages were identified based on peel color index which also showed significant differences in physicochemical properties and starch granule morphology. The effect of physical properties of digesta was evaluated by monitoring the viscosity throughout the simulated digestion process and comparing two different physical structures of banana: (1) unhomogenized cut samples which have intact tissue structure and (2) homogenized slurry representing disrupted cellular structure. During ripening process, a decrease in starch content was noted with a concomitant formation of sugars and increasing concentration of acids. Green unripe stages showed the highest rate of starch hydrolysis in both physical structures and a decreasing trend was observed as ripening proceeded. The high digesta viscosity values of ripe stages was found to have an inhibitory effect on starch hydrolysis. Similarly, the differences in physical structure of food affected the digestive enzymes efficiency in breaking down starch. These results suggested that the physicochemical changes accompanying maturation and the physical properties (i.e. high viscosity and presence of intact cell structure) of food could significantly impact the rate of starch digestion.

## Introduction

‘Saba’ [*Musa ‘saba’(Musa acuminata* × *Musa balbisiana)*] is one of the banana cultivars considered to have good potential in the export industry. The fruit crop is usually grown in Southeast Asia with local names such as *giant pisang kepok* (Indonesia), *pisang abu nepah* (Malaysia), *kluai hin* (Thailand), *chuoi mat* (Vietnam), and Saba or *cardaba* (Philippines)^1^. In the Philippine setting, this variety is the most important among the many banana cultivars in terms of commercial production and trade because of its wide range of applications in the domestic market. Most people in the rural areas regarded Saba banana as one of their food sources often consumed as an alternative food staple to rice and corn. The fully ripe fruit can be eaten raw but is more commonly cooked by boiling and frying in syrup^2^. Likewise, the green unripe stage is consumed like a vegetable in making savory dishes and can be processed into several starch-based food products such as chips, ketchup, and sauces. Due to its more affordable price when compared to dessert bananas and high possibility of formulation into a variety of products, an increase in both consumption and utilization has been observed over the year^3^.

Aside from being a staple food and as a raw material in various processed products, banana, in general, is known to offer great health benefits and nutritional value^4,5^. In the green stage, bananas stand out for having high resistant starch contents^6^. Substantial researches had been conducted linking resistant starch in enhancing colonic health, acting as a vehicle to increase total dietary fibre content of food, and aiding diabetes and weight management^7^. Conversely, previous studies also suggested the restriction of banana as desserts or snacks as it contains approximately twice the amount of carbohydrate compared to other fruits. The conversion of starch into free sugars during the ripening process may influence its postprandial blood glucose response^8^. Thus, studying the digestibility of banana, particularly its starch content, at different maturity stages is necessary to understand its nutritional implications and functionality as an energy source.

Research and development efforts on banana have been ongoing. To date, information on digestibility of banana starch and food products with banana flour as a raw material is extensive. Jiang *et al*.^9^ reported that the starch from different banana cultivars showed differences in structural, physicochemical, and digestibility characteristics. Bi *et al*.^10^ concluded that the digestibility of green banana flour and starch was affected by chemical composition, particularly pectin, and granule morphology, crystalline structure, amylose content, branched chain length distributions of amylopectin, and starch molecular weight. However, little to no information is available on the digestibility of fresh banana fruit, specifically the Saba banana variety, and the influence of maturation on its starch digestibility. When assessing the rate of starch digestion, it is also essential to elucidate the food matrix effects on the extent by which the food component is hydrolyzed. The complex food matrix of fresh Saba banana may influence the catalytic efficiency of digestive enzymes in breaking down starch, and thus affecting its bioavailability. One way to establish the structure of such matrix is through food processing or oral mastication, with or without disruption of the tissue structure. This can have a critical role on starch digestibility by affecting the accessibility of digestive medium to starch in the food or through its influence on the surface area available for digestion^11^. As reported in previous *in vivo* and *in vitro* studies about almonds^12^, legumes^13^, carrots^14^, and rice^15^, intactness of cell wall structure could be a controlling factor in the bioaccessibility of nutrients from plant-based foods. For these reasons, a simulated gastrointestinal *in vitro* digestion model was employed to examine the digestibility of starch in Saba banana as affected by maturity and physical characteristics of the digesta, which involved viscosity and physical structure. The impact of viscosity and gross structure on starch digestibility was evaluated through preparation of unhomogenized cut and homogenized slurry samples. The former represented minimally processed food product which retained the tissue structure and could also be as a result of limited comminution. The latter, on the other hand, represented structure-less food materials as a result of processing approaches such as crushing and blending or thorough mastication that fully disrupt the cellular structure.

## Results and Discussion

### Physicochemical properties

Saba banana, same as other climacteric fruits, is harvested at mature green stage and then allowed to ripen naturally or artificially. One of the most noticeable changes during ripening process is in the peel color which acts as a simple indicator of fruit maturity. During ripening of Saba banana, lightness (L*) values slightly increased while hue angle (h°) significantly decreased as the peel changed from mature green in unripe stage to brownish-yellow color in ripe stages (Table 1). The presence of brown flecks at the last stage resulted to a further drop in the values of h° and L*. No definite trend in the values of chroma (C) means that during ripening, different colors are present simultaneously since the green color of chlorophyll pigment is degraded and at the same time the characteristic yellow color of carotenoid is synthesized^16^, coupled with a build-up of diverse types of anthocyanins^17^. The enzyme, chlorophyllase, is responsible for the breakdown of chlorophyll which is known to increase in activity on the rise of climacteric phase^4^.

**Table 1:** Physicochemical properties of five maturity stages of Saba banana.

Maturity	MC (%)	TSS (°Brix)	pH	TA (% malic acid)	Gluc (%)	Fruc (%)	Suc (%)	RS (%)	Total starch (%)	L*	C	h°
1	62.31 ± 0.19^c^	4.20 ± 0.40^e^	6.66 ± 0.02^a^	0.037 ± 0.003^e^	0.22 ± 0.03^c^	0.22 ± 0.03^c^	2.34 ± 0.34^c^	17.37 ± 0.57^a^	26.05 ± 0.13^a^	52.19 ± 2.67^c^	43.75 ± 3.71^a^	105.73 ± 1.79^a^
2	62.29 ± 0.32^c^	5.15 ± 0.23^d^	6.41 ± 0.02^b^	0.043 ± 0.004^d^	0.39 ± 0.02^c^	0.39 ± 0.01^c^	2.55 ± 0.16^c^	17.08 ± 0.64^a^	24.28 ± 0.94^b^	56.38 ± 2.00^ab^	38.33 ± 2.09^b^	99.84 ± 2.38^b^
3	62.27 ± 0.24^c^	7.80 ± 0.26^c^	5.89 ± 0.03^c^	0.060 ± 0.001^c^	1.87 ± 0.46^b^	2.15 ± 0.69^b^	4.68 ± 0.89^b^	14.00 ± 0.51^b^	21.82 ± 0.94^c^	57.50 ± 2.93^a^	42.22 ± 6.14^ab^	93.21 ± 1.99^c^
4	63.19 ± 0.35^b^	11.70 ± 0.40^b^	4.96 ± 0.01^d^	0.157 ± 0.003^b^	3.57 ± 0.59^a^	3.98 ± 0.63^a^	5.35 ± 0.91^b^	7.61 ± 0.13^c^	15.49 ± 0.40^d^	57.69 ± 0.80^a^	37.81 ± 1.05^b^	80.83 ± 1.23^d^
5	64.33 ± 0.27^a^	16.45 ± 0.23^a^	4.70 ± 0.01^e^	0.218 ± 0.002^a^	3.65 ± 0.37^a^	3.96 ± 0.37^a^	7.99 ± 0.91^a^	5.05 ± 0.40^d^	14.75 ± 0.61^d^	53.81 ± 2.67^cb^	38.48 ± 3.50^ab^	77.93 ± 1.17^e^

Moisture content (MC), titratable acidity (TA), glucose (Gluc), fructose (Fruc), sucrose (Suc), resistant starch (RS), and total starch are reported as fresh weight (%). Lightness (L*) is from 0 for black to 100 for white; chroma (C) is calculated as C = (a*^2^ + b*^2^)^1/2^; and hue angle (h°) is calculated from the arctangent of b*/a*. Values are means ± standard deviations of three replicates except for moisture content, n = 7, color, n = 8, and resistant and total starch, n = 4. Mean values with different letters in the same column indicate significant differences (p < 0.05).

Aside from color, other physicochemical properties used to confirm the stages of banana were pH, TA, moisture content, total soluble solid (TSS), and sugar and starch contents. With the formation of organic acids, specifically malic and citric acids, pH and titratable acidity decreased and increased, respectively, during ripening^2^. The moisture content was found to increase (Table 1) during ripening due to respiratory breakdown of starches and osmotic transfer from peel to pulp^18^, which could be attributed to sugar accumulation in the pulp. The formation of sugar was manifested in the increasing value of TSS as ripening occurred as well as the changes in sugar contents. Sucrose, glucose, and fructose are the most common sugars accumulated during fruit development and ripening^5^. In this study, it was evident that sugars were present even at initial stage of ripening. Although no significant changes in sugar contents of stages 1 and 2 were detected, the concentration was found to be increasing and became significant at the onset of stage 3. For all stages, sucrose was found in the greatest amount with more than 3-fold increase in concentration from stage 1 to 5. In contrast, from around 0.2% in the unripe stage, glucose and fructose increased by more than 10-fold at the last stage. The increased percentage of sugars in banana pulp during fruit ripening was primarily due to degradation of starch^19^.

Starch is the principal component of mature green bananas, which undergoes important chemical changes during postharvest ripening^19–21^. The total starch content of Saba banana showed a reduction of more than 40% from stage 1 to 5 which was comparable to the results of previous studies. Mohapatra *et al*.^18^ reported that in triploid hybrid group of bananas, starch content decreased from 20–30% to 1–2% during ripening. Moreover, Aquino *et al*.^19^ studied starch contents of different banana varieties including one from ABB genomic group, in which Saba banana is categorized, and showed starch contents of 27.93% and 13.33% for unripe and ripe stages, respectively. Though the pattern of starch degradation may vary significantly among banana varieties, soluble sugars, mainly sucrose, accumulate following the starch breakdown during ripening^22^. In the present study, more than 70% of resistant starch was rapidly degraded enzymatically at the end of storage. The transformation of starch to sugars is catalyzed by the increased activity of several starch degrading enzymes during ripening such as alpha- and beta-amylases, phosphorylases, and debranching enzymes^4,5,21,23^.

### Sugar contents and their behaviors during simulated digestion

**Figure 1:**
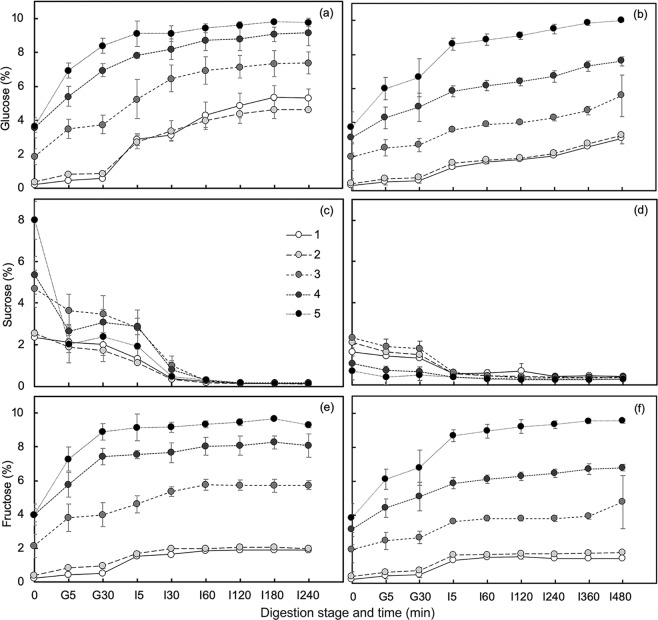
Release behaviors of sugars (%) in different maturity stages of slurry (**a**,**c**,**e**) and cut (**b**,**d**,**f**) Saba banana during simulated digestion. Error bars represent standard deviation (*n = *3).

The study observed the changes in sugar contents of homogenized slurry and unhomogenized cut Saba banana during simulated *in vitro* digestion in terms of glucose, sucrose, and fructose concentrations (Fig. 1). The variations in the structure of slurry and cut samples showed an effect in the sugar profile even at the initial phase of simulated digestion. From 0 to the end of gastric digestion, an increase in the concentrations of glucose and fructose while a sharp decrease in sucrose content were observed in all maturity stages. Glucose (Fig. 1a) and fructose (Fig. 1e) of slurry samples were found to increase ranging from 97–165% and 89–147%, respectively, in stage 1 to 5. This trend was also noticed in cut samples (Fig. 1b,f); however, comparing from slurry counterpart, lesser increment was observed with percent difference values ranging from 40–96% in glucose and 10–88% in fructose. Sucrose, on the other hand, showed a percent decrement ranging from 17–69% in slurry (Fig. 1c) and 19–36% in cut (Fig. 1d). During the gastric conditions, acid catalyzes the hydrolysis of sucrose chemically^24^. The mechanism behind acid-catalyzed sucrose hydrolysis involves protonation to the oxygen of the glycosidic bond followed by fructosyl-oxygen bond cleavage forming D-glucose and a fructose carboxonium ion, which can react with water to form D-fructose^25^. The varying contents of sugars between slurry and cut samples could be due to their different physical structures; the former having damaged or disrupted cells brought about by homogenization process while the latter had intact tissue/cell structure. The disrupted cells in slurry samples facilitated the immediate release of sugars making them more available for reaction or hydrolysis, whereas in cut samples, sugars are enclosed in cell walls which may possibly resist acid disruption in the gastric phase of digestion.

The addition of small intestinal enzymes such as invertase, which is responsible for the breakdown of sucrose to glucose and fructose monosaccharide units in the intestinal phase of digestion^26^, showed more prominent changes in the sugar contents of slurry and cut Saba banana. More than 94% of sucrose was hydrolyzed in all stages of slurry sample. In contrast, after 240 min of intestinal digestion, cut samples exhibited a lower percent decrement in sucrose concentration ranging from 63–85% (stage 1 to 5). Almost the same values were observed when cut samples continued until 480 min of intestinal digestion. The lower percent decrement could be accounted to slow release rate of sugars and hindered penetration of digestive enzymes into the intact tissue of the sample. Based on the initial and final values of glucose and fructose, the green mature Saba banana (stage 1) showed the highest percent increment in both slurry (25-fold in glucose and 8.5-fold in fructose) and cut (10-fold in glucose and 6.5-fold in fructose) states, and a decreasing trend was observed at increasing maturity. Aside from sucrose, starch is also hydrolyzed which contributed to the increased glucose accumulation during the simulated intestinal phase of digestion.

### Combined effect of maturity and physical properties of digesta on starch hydrolysis

**Figure 2:**
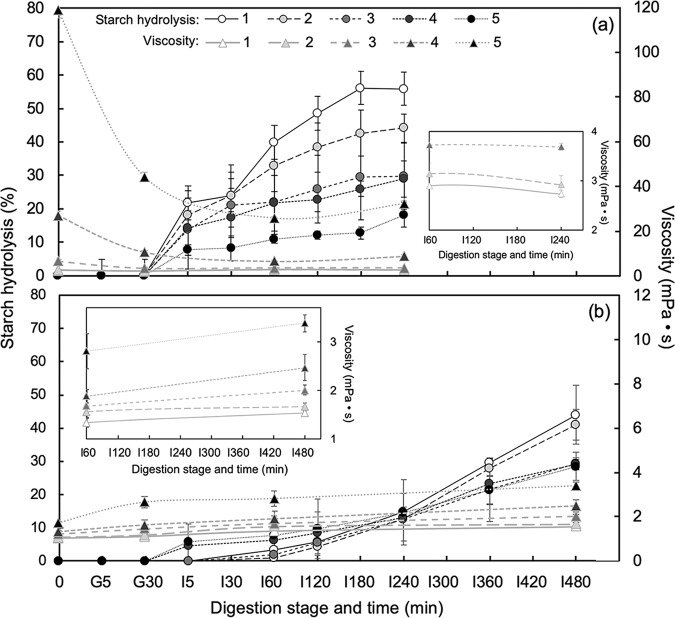
Starch hydrolysis (%) and viscosity of digesta (mPa·s) in different maturity stages of slurry (**a**) and cut (**b**) Saba banana during simulated digestion. Error bars represent standard deviation (*n* = 3).

A significant decrease in starch hydrolysis (%) was observed in both homogenized slurry and unhomogenized cut samples as Saba banana proceeded ripening (Fig. 2). The green unripe stages (stage 1 and 2) showed the highest starch hydrolysis with values of 56% and 44%, respectively, for slurry samples (Fig. 2a) after 240 min of simulated digestion. Lesser values were obtained in cut samples (Fig. 2b) with 44% and 40% starch hydrolysis for stages 1 and 2, respectively, even at a longer digestion time of 480 min. The last stage was found to have the lowest starch hydrolysis (%) but was not significantly different from stages 3 and 4. The values of ripe stages (stage 3, 4, and 5) varied ranging from 18–30% in slurry samples while cut showed a hydrolysis value of around 30% (after 480 min of intestinal digestion). Low percent hydrolysis was obtained in cut samples at intestinal digestion time of 240 min which ranged from 12–15% with stage 1 having the highest value. The interplay of different factors in fresh Saba banana could account for the decreasing rate of starch hydrolysis as the fruit ripens. One factor that contributed to the outcome was the differences in the physical characteristics of the digesta. Prior to simulated *in vitro* digestion, digesta viscosity of Saba banana showed increasing values with increase in fruit maturity. However, as the homogenized slurry passed through the successive stages of the simulated gastrointestinal process, there was a progressive decrease in the viscosity values of the ripe stages. A percent decrement of 73%, 67%, and 3% was observed in viscosity values of stages 5, 4, and 3, respectively, after simulated digestion. This trend could be brought about by the dilution of samples upon addition of gastric and intestinal fluids, possible effect of severe acidic conditions on the digesta, and enzymatic liquefaction of starch. In contrast, all maturity stages of unhomogenized cut samples showed increasing viscosity values due to the continuous release of food components during the simulated digestion process. Nevertheless, the trend of increasing viscosity as fruit ripening proceeded was consistent even after 4 and 8 hr of intestinal digestion for slurry and cut samples, respectively. Though this may be accounted to the different dilution values applied to each stage in order to come up with initial digesta having the same starch content; however, increasing viscosity may also be explained by the presence of water-soluble pectins in the samples. A number of reports showed the conversion of water-insoluble pectin to water-soluble pectin during fruit ripening^27^. Hence, increasing amounts of water-soluble pectins were observed in fruits such as guava^28^, cherry tomatoes^29^, peach^30^, plums^31^, and banana^27^ during maturity storage. This was further confirmed by the study of Lustre *et al*.^2^ which reported increasing pectin content (as calcium pectate) in naturally and chemically-ripened Saba banana pulp during storage.

Water-insoluble pectin, commonly known as protopectin, is the form of pectic substance found in immature fruit. As the fruit ripens, enzymes convert protopectin to a more water-soluble pectin and the texture becomes soft. It is this pectin from ripe fruit that is capable of swelling in water and form gel^32^. The ability to form gels in the presence of sugar and acid and viscosity build-up are the most unique and outstanding properties of pectin^33,34^. In the study, the increasing sugar and acid during ripening could trigger the gelling reaction with pectin resulting to an increase in viscosity of the digesta, and thus low rate of hydrolysis in the ripe stages of Saba banana. The inhibition of propulsive and mixing effects, which results to less frequent interactions between substrates and digestive enzymes, is the main impact of increased viscosity on starch digestibility^35^. It was found that the high resistant starch content of unripe stages of Saba banana had little or no effect on starch digestibility due to the inverse relationship of maturity and starch hydrolysis rate. Though resistant starch contributes to the proportion of starch that escapes from digestion in the small intestine and seems to reduce the accessibility of the substrates to amylases^23^, this type of starch lacks gel-forming effect which is present in non-starch polysaccharides like pectin^36^. Therefore, the physical properties of digesta is also a factor that could exhibit an impact on starch digestion rate.

The presence of pectin could also significantly reduce the rate of digestion by its association with starch components and digestive enzymes. The interaction of pectin with amylose and its protective effect on starch swelling and enzymatic hydrolysis were cited as the possible reasons for the increased concentrations of slowly digestible and resistant starches in corn starch, respectively^37^. Moreover, an association between pectin and amyloglucosidase, was observed which changed the digestive enzyme’s conformation, and thus hindered its access to starch^38^.

**Figure 3:**
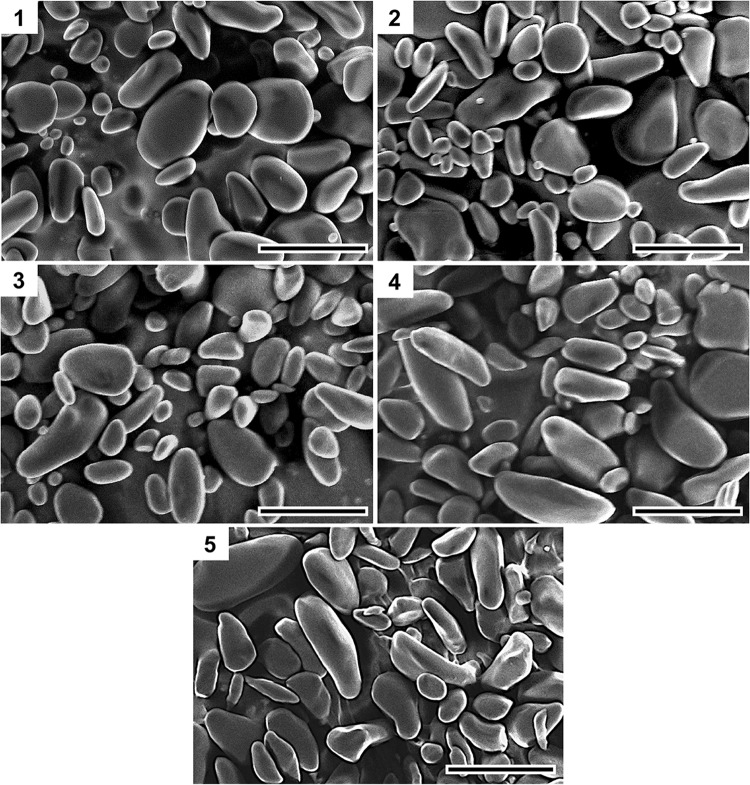
Morphological observation of starch granules from different maturity stages of Saba banana. Scale bar = 50 µm.

The morphological structure of starch granule could also influence digestibility^39,40^. Scanning electron micrographs of Saba banana starch granule showed small and large spherical and elongated starch granules with lenticular shape for unripe stages (Fig. 3). As maturity advanced, a decrease in the number of small granules was observed and starch was contorted to an elongated structure which was apparent in the last stage of maturity. These findings were similar to those found by Soares *et al*.^41^ which reported that starch granules of dessert bananas were predominantly small and leaf-like while plantain had both small and elongated granules at the green unripe stage. As a result of starch degradation during ripening, the small granules in plantain disappeared but the elongated shape of starch granules remained in both types. This particle size and shape of starch may also play an important role for digestibility as small and round granule size have been reported to have higher enzymatic susceptibility^22,42^.

Additionally, it has been reported that food structure is one of the major determinants of the rate of starch digestion^43–45^. The physical structure could be an intrinsic factor that hindered the access of digestive enzymes and slow down the degradation of starch^36^ from the food matrix of Saba banana. Therefore, modifications in its structure and composition through processing might be reasonably expected to have a significant effect on starch digestibility. The same condition applies during the oral phase of digestion, wherein mastication could break down the particles of food into small pieces. The process increases the surface area of food particles, promoting the ingress of digestive enzymes and liberating starch that is then rapidly digested. However, there are limits to the capacity of humans to reduce the particle sizes of foods during mastication^13^ and no endogenous enzymes present in the human upper gut can degrade plant cell walls^14^. Thus, depending on the initial of structure of food^45^, the degree of mastication^44^, and the particle size of food materials^13^, lesser physical degradation (i.e. incomplete comminution and larger particles) occurring during oral digestion could increase the probability of survival of some cells with structures still remained intact and components encapsulated by cell walls. When cells are not thoroughly disrupted, the intact cell wall could act as barrier to decomposition during gastric and small intestine passage, and thus the starch that remained inside the structure is not immediately hydrolyzed. The only way enzymes could penetrate the food is by diffusion through the intact cell walls; however, this penetration effects of digestion is relatively a slow process^11^. Consistent with this expectation, in this study, it was found that green mature Saba banana (stage 1 and 2) of slurry had significantly higher starch hydrolysis at a short intestinal digestion time of 240 min than cut samples which were digested at 480 min. This trend was also observed in the ripe Saba banana samples (stage 3, 4, and 5) when compared at the same digestion time of 240 min (18–30% for slurry and 13–15% for cut). The extension in the digestion time of cut samples to 480 min brought an increment of 14%, 16%, and 17% in stages 5, 4, and 3, respectively. This increase in starch hydrolysis may also be observed in slurry samples if digestion time was extended; however, the possibility of the effect of high digesta viscosity would limit the enzyme accessibility to starch polymers, and thus may possibly result to a low hydrolysis value.

## Conclusion

The combined effect of physical properties and physicochemical changes during maturation could account to the variations in starch digestibility of different stages of Saba banana. Among the physical properties discussed in this study that significantly affect digestibility were viscosity and physical structure. The observed increase in viscosity could be attributed to food component that is capable of forming a gel, such as water-soluble pectin, and its interaction with water, sugar and acid, which all have shown to increase as the fruit proceeds ripening. The differences in physical structure could correspond to the degree of particle breakdown during mechanical processing or oral mastication. Highly viscous digesta was found to have low impact on starch digestibility due to immobilization of digestive components. Same effect was observed in food samples with intact cell structure as this hindered the action of enzymes. Both high viscosity and presence of intact cell wall may offer physiological advantages in preventing the sudden surge in *in vivo* postprandial blood glucose level upon ingestion of banana. However, this study needs further investigation before conclusive evidence can be obtained as various factors are synergistically involved in the starch digestibility of this variety of banana.

## Methods

### Materials and chemicals

**Figure 4:**
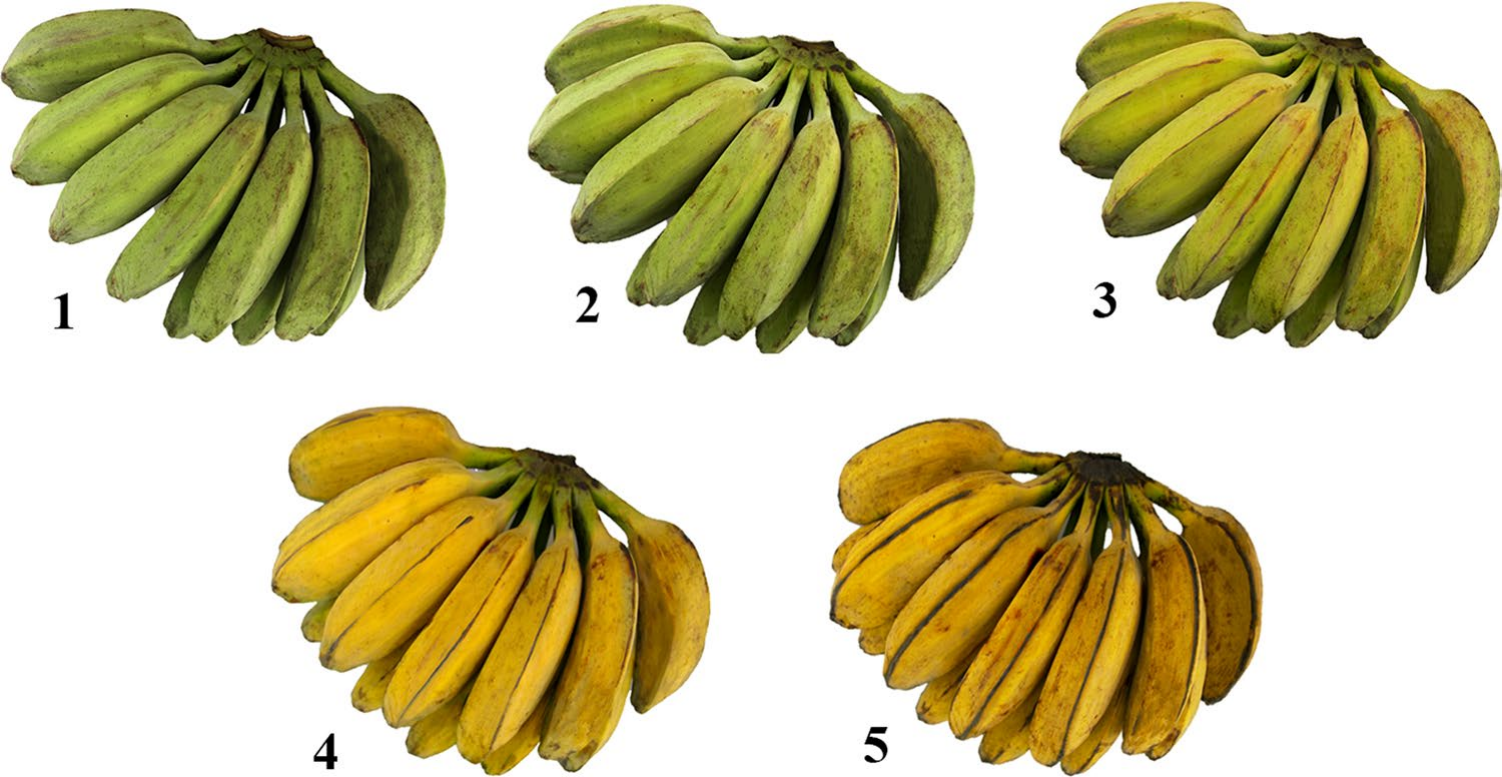
Five maturity stages of Saba banana used in the study.

Commercial green mature Saba bananas were purchased from Diamond Star Agro-Products Inc., Taguig City, Philippines. The fruit was allowed to ripen until 5 different maturity stages developed as shown in Fig. 4 — all green (stage 1), green but turning yellow (stage 2), greenish yellow (stage 3), yellow with green tips (stage 4), and yellow with brown flecks (stage 5). Enzymes used in simulated digestion such as pepsin (porcine gastric mucosa, >250 U mg^−1^ solid), pancreatin (hog pancreas, 4x USP), and invertase (grade VII from baker’s yeast, >300 U mg^−1^ solid were bought from Sigma-Aldrich (St. Louis, Mo., U.S.A.). Amyloglucosidase (3260 U mL^−1^) and resistant starch assay kit (K-RSTAR) were purchased from Megazyme International (Wicklow, Ireland). Sugar standards (sucrose, glucose, and fructose) and ultrapure water were supplied by Wako Pure Chemical Industries (Osaka, Japan).

### Sample preparation

A minimum of 7 Saba banana fingers from 6 bunches were randomly selected every sampling period. The samples were peeled, cut into small round sections, and divided into three portions. One part was freeze-dried (FDU-1100, Eyela, Tokyo, Japan), ground, and passed through a 0.5 mm mesh sieve (Sanpo, Tokyo, Japan) before analysis of starch content. Another portion was allotted for the simulated digestion. The slurry state was homogenized using a household blender (NM200, Yamazen, Tokyo, Japan) for 2 minutes while cut sample (with dimension of ca. ≤3 mm) was prepared using a combination of manual cutting and food chopper (Tefal, Rumilly, France). Both physical states were mixed with water to have the same initial starch content of approximately 4%. The remaining raw Saba banana samples were used for the determination of pH, titratable acidity (TA), and total soluble solids (TSS) using supernatant from homogenized pulp (10 g banana pulp:30 mL distilled water).

### Physicochemical analyses

Physicochemical analyses were used as indices to determine the maturity of Saba banana. Peel color was assessed by capturing an RGB image using a digital camera (E-510 Olympus, Tokyo, Japan) with 1/125 shutter speed, F4.0 exposure, and ISO 100. The captured RGB image was converted to CIE L*a*b* to obtain L* (lightness), a* (–greenness to +redness), and b* (–blueness to +yellowness) values using Adobe Photoshop CC (Adobe, San Jose, CA, U.S.A.). Chroma [C = (a*^2^ + b*^2^)^1/2^] and hue angle [h° = tan^–1^(b* /a*)] were calculated from the chromaticity coordinates. Moisture content was analyzed by gravimetric heating using an oven dyer (Oven 8150, Labserv, Longford, Ireland) following the method of AOAC^46^ with slight modification. pH, TSS, and TA was determined using the protocols of Dadzie & Orchard^20^ with minor modifications. A pH meter (AS800, As One, Osaka, Japan) and a digital refractometer (PR-101, Atago, Tokyo, Japan) were used to measure pH and TSS, respectively, while TA was obtained by titration to a pH of 8.1 and was expressed as malic acid (%).

### Sugar analysis

A reversed-phase high-performance liquid chromatography (RP-HPLC) was used for the separation and determination of sugars (sucrose, glucose, and fructose) in the digested fractions. The supernatants collected from simulated digestion were first filtered through 0.45 µm membrane filter (Advantec, Tokyo, Japan). The chromatographic separation was performed using HPLC system (Shimadzu, Kyoto, Japan) equipped with pump (LC-20 AD), refractive index detector (RID-20A), column (Shim-pack SCR-101N), oven (CTO-20 AC), and degassing unit (DGU-20A3). The mobile phase, ultrapure water, was pumped at a flow rate of 0.8 mL min^−1^ under isocratic elution at a constant oven temperature of 60 °C. Analytical data were collected and processed by LabSolutions software (Shimadzu, Kyoto, Japan).

### Total and resistant starch analysis

Total starch (TS) and resistant starch (RS) contents were determined using an assay kit^47^ following AOAC Official Method 2002.02. Briefly, 100 ± 5 mg of freeze-dried sample was added with 4 mL pancreatic α-amylase (10 mg/mL) containing amyloglucosidase (3 U/mL). The sample was incubated at 37 °C with continuous shaking (200 strokes/ min) for exactly 16 h to allow solubilization of non-resistant starch and hydrolyzation to D-glucose. After incubation, the reaction was terminated by addition of 4 mL ethanol (99% v/v) and the supernatant was separated with the pellet by centrifugation at 1500 × *g* for 10 min. The resistant starch in the pellet was re-suspended twice in 8 mL of 50% ethanol and centrifuged again at 1500 × *g* for 10 min. The recovered pellet was then dissolved in 2 M KOH for approximately 20 min in an ice-water bath with vigorous stirring. The solution was neutralized with 8 mL of 1.2 M sodium acetate buffer (pH 3.8) followed by addition of 0.1 mL of amyloglucosidase (3,300 U/mL) and incubation at 50 °C for 30 min with intermittent mixing. The volume of the sample was adjusted to 100 mL with distilled water. An aliquot of the solution was centrifuged at 1500 × *g* for 10 min. Free glucose from resistant starch was determined using 0.1 mL aliquots (in duplicate) of diluted supernatant and 3 mL glucose oxidase/peroxidase (GOPOD) reagent after incubation at 50 °C for 20 min. Absorbance of the sample and standard (D-glucose) were measured at 510 nm in UV-Vis spectrophotometer (V-630Bio, Jasco, Tokyo, Japan) against a reagent blank consisting of 0.1 mL of distilled water. For the determination of non-resistant starch, the supernatants obtained from washings were pooled and adjusted to 100 mL with 100 mM sodium acetate buffer (pH 4.5). An aliquot (0.1 mL) was incubated with 10 μL of diluted AMG solution (300 U/mL) in 100 mM sodium maleate buffer (pH 6.0) for 20 min at 50 °C. GOPOD reagent (3 mL) was added and further incubation of the sample was done at 50 °C for 20 min. Absorbance of free glucose from non-resistant starch was measured the same method as resistant starch. Resistant and non-resistant starches were calculated by multiplying the measured free glucose using a conversion factor of 0.9 (factor to convert from free D-glucose, as determined, to anhydro-D-glucose as occurs in starch), which is shown below:

$${\rm{Resistant}}\,{\rm{starch}}\,{\rm{or}}\,{\rm{Non}}-{\rm{resistant}}\,{\rm{starch}}\,( \% )=\Delta {\rm{E}}\times \frac{{\rm{F}}}{{\rm{W}}}\times {\rm{V}}\times 0.9$$

where $\Delta {\rm{E}}$ is the absorbance of the reaction against the reagent blank, F is the conversion from absorbance to micrograms (100 µg of D-glucose divided by the GOPOD absorbance for the 100 µg of D-glucose), W is the dry weight of sample analysed, and V is the volume of extract.

### Simulated *in vitro* gastrointestinal digestion

A two-stage simulated *in vitro* gastrointestinal digestion model was employed following the method of Dartois *et al*.^48^ with modifications by Thuengtung *et al*.^15^. Fresh Saba banana in each maturity stage (170 g) were directly transferred into a jacketed-glass reactor maintained at 37 ± 1 °C with continuous stirring. Simulated gastric digestion was initiated by adjusting the pH to 1.2 ± 0.1 using different molar concentrations of HCl and addition of simulated gastric fluid containing 0.12 g pepsin and buffer with 0.2% (w/v) NaCl (pH adjusted to 1.2). After 30 min of gastric digestion, the pH was changed to 6.8 ± 0.1 by addition of different molar concentrations of NaOH to deactivate pepsin and simulate the digestion in the small intestine. The intestinal digestion process began when the simulated intestinal fluid, containing 0.1 g pancreatin, 7.5 mg invertase, 2 mL amyloglucosidase, and buffer with 0.68% (w/v) monobasic potassium phosphate (KH_2_PO_4_) (pH adjusted to 6.8), was added to the reaction mixture. Aliquots of supernatants were collected from the following time periods: after 5 (G5) and 30 min (G30) of gastric digestion; and after 5 (I5), 30 (I30), 60 (I60), 120 (I120), 180 (I180), and 240 min (I240) of small intestinal digestion for slurry samples while cut samples was continued until 480 min (I480). The digested fractions were mixed with 3 mL of 95% ethanol to stop the enzymatic reactions, centrifuged at 1800 × *g* for 10 min, and stored at −20 °C until further analysis.

### Digesta viscosity measurement

Viscosity of the supernatant from digesta was measured using a torsional oscillation viscometer (Viscomate VM-10A, Sekonic, Tokyo, Japan). Four sampling points were selected during the course of *in vitro* digestion representing initial value at time 0, after gastric digestion (G30), during intestinal digestion (I60), and at the end of digestion (I240 and I480 for slurry and cut samples, respectively). The supernatant was withdrawn from the digestion reactor and viscosity was measured under static conditions at 37 ± 1 °C.

### Total starch and starch hydrolysis

The total starch (TS) content from the freeze-dried Saba banana is defined as follows:

$${\rm{TS}}\,( \% )={\rm{Resistant}}\,{\rm{starch}}+\left[{\rm{Non}}-{\rm{resistant}}\,{\rm{starch}}-\left({\rm{Initial}}\,{\rm{Glucose}}+\frac{{\rm{Initial}}\,{\rm{Sucrose}}}{2}\right)\right]$$

The starch hydrolysis (%) was computed based on the amounts of sucrose, glucose, and fructose accumulated during *in vitro* digestion. It was estimated by deducting the initial glucose content of the sample and the glucose hydrolyzed from sucrose. The latter was expected to be equal to the amount of fructose formed during sucrose hydrolysis and the value was deducted to the total amount of glucose accumulated. Thus, the formula is defined as follows:

$${\rm{Starch}}\,{\rm{hydrolysis}}\,( \% )=\frac{({\rm{Glucose}}-{\rm{Glucose}}\,{\rm{at}}\,{\rm{G}}30)-({\rm{Fructose}}-{\rm{Fructose}}\,{\rm{at}}\,{\rm{G}}30)}{{\rm{TS}}}\times 100$$

### Scanning electron microscopy

Banana starch was obtained using the extraction method described by Espinosa-Solis *et al*.^39^ with some modifications. Samples were fixed on aluminium stubs using double-sided tape and viewed under a scanning electron microscope (SEM) (SU1510, Hitachi high-technologies, Tokyo, Japan) using an acceleration voltage of 15 kV.

### Statistical analysis

The results were presented as mean values ± standard deviation. The data were analyzed using one-way analysis of variance and the means of results for each experiment were compared using Tukey’s test (*p* < 0.05). Statistical analyses were run using R software version 3.5.2^49^.

### Ethical approval

This article does not contain any studies with human participants or animals performed by any of the authors.

## Data Availability

The research data of this study will be provided upon request.
